# Single-cell transcriptomics for immune profiling of cerebrospinal fluid in neurological diseases

**DOI:** 10.3389/fimmu.2025.1599303

**Published:** 2025-05-29

**Authors:** David Ramos-Vicente, Paola Monterosso, Oriol de Fàbregues, Gerard Roch, Miquel Vila, Jordi Bové

**Affiliations:** ^1^ Neurodegenerative Diseases Research Group, Vall d’Hebron Research Institute (VHIR)-Network Center for Biomedical Research in Neurodegenerative Diseases (CIBERNED), Barcelona, Spain; ^2^ Movement Disorders Unit, Neurology Department, Vall d’Hebron University Hospital, Barcelona, Spain; ^3^ Catalan Institution for Research and Advanced Studies (ICREA), Barcelona, Spain; ^4^ Department of Biochemistry and Molecular Biology, Autonomous University of Barcelona, Barcelona, Spain; ^5^ Aligning Science Across Parkinson’s (ASAP) Collaborative Research Network, Chevy Chase, MD, United States

**Keywords:** neuroinflammation, neuroimmune, neurodegenerative disease, neurological infections, brain metastases, lymphocyte, innate immune, single-cell omics

## Abstract

In this comprehensive review, we delve into the significant body of research on single-cell transcriptomics in cerebrospinal fluid (CSF) to understand neurological diseases with autoimmune, neurodegenerative, infectious, or oncogenic origins. We thoroughly examine all published studies in these areas, with a particular focus on multiple sclerosis, Alzheimer’s disease, and Parkinson’s disease. For these diseases, we review findings related to immune cells that infiltrate the brain, based on postmortem brain tissue analyses and include CSF cytometry findings. Single-cell RNA sequencing (scRNA-seq), single-cell T cell receptor sequencing (scTCR-seq), and single-cell B cell receptor sequencing (scBCR-seq) are increasingly vital tools for studying CSF to understand various aspects of neurological diseases. These advanced techniques allow researchers to explore the etiopathogenesis of these conditions by identifying the roles and interactions of different immune cells. scRNA-seq provides detailed insights into the gene expression profiles of individual cells, revealing how specific cell types contribute to disease progression. scTCR-seq and scBCR-seq enable the study of clonal expansion in T and B cells, respectively, and facilitate antigen prediction, helping to uncover the nature of antigens that trigger adaptive immune responses. By integrating these technologies, scientists can define new therapeutic targets and categorize patients, leading to more personalized and effective treatments. This review highlights the promising advancements and addresses the current limitations of single-cell transcriptomics in the context of CSF and neurological diseases, setting the stage for future breakthroughs.

## 1 Introduction

scRNA-seq data analysis has revolutionized our understanding of cellular heterogeneity and gene expression dynamics at an unprecedented resolution. scRNA-seq allows for the analysis of gene expression profiles at the individual cell level. The process involves isolating single cells, lysing them, and reverse transcribing their RNA into complementary DNA (cDNA), which is then amplified to create libraries for high-throughput sequencing (1). Methodologies for scRNA-seq include droplet-based microfluidics, microwell-based methods, and plate-based techniques (2–4). Lymphocyte receptor sequencing adds a new layer of information to scRNA-seq, allowing for a deeper study of the adaptive immune response (5). Single-cell T cell receptor sequencing (scTCR-seq) enables the analysis of TCR diversity and specificity at the single-cell level by isolating individual T cells, amplifying their TCR alpha and beta chain transcripts, and sequencing them. Similarly, single-cell B cell receptor sequencing (scBCR-seq) is used to analyze B-cell receptor (BCR) diversity and specificity. Integrating scTCR-seq and scBCR-seq with scRNA-seq provides a multidimensional perspective on immune cell diversity, gene expression profiles, and antigen specificity. This combined approach enhances our understanding of cellular functions, interactions, and the mechanisms underlying adaptive immune responses in different diseases (6, 7).

Various bioinformatic tools enable a wide range of analyses, providing unique insights into cellular functions and interactions (8–10). Developmental trajectories and RNA velocity analyses allow researchers to infer the dynamic processes of cell differentiation and lineage commitment. Cell-cell communication studies reveal intricate signaling networks between different cell types, enhancing our understanding of tissue organization and function. Gene regulatory networks can be reconstructed to identify key transcription factors and regulatory elements controlling gene expression. Multimodal analyses, integrating protein expression, chromatin accessibility, transcription factor-binding sites, and expression quantitative trait loci (eQTL), offer a comprehensive view of cellular states and regulatory mechanisms (11, 12). Bioinformatics tools utilizing TCR sequences enable the prediction of antigens, holding the potential to elucidate the specific antigens that trigger adaptive immune responses in various diseases (13, 14).

CSF scRNA-seq and scTCR-seq and scBCR-seq, are providing new insights into the roles of both innate and adaptive immune responses in the etiopathogenesis of neurological diseases of distinct origins (15–17). These advanced technologies are elucidating the complex interactions between different immune cell subsets and their contributions to disease progression and therapeutic outcomes (18–20). In addition, antigen prediction followed by experimental validation should shed light in the near future on the etiopathogenesis of neurological diseases with unknown etiology (21). Together, these approaches are enhancing our understanding of immune mechanisms and beginning to facilitate the stratification of patients, paving the way for more personalized and effective treatments with reduced side effects.

In this review, we will discuss all the peer-reviewed published CSF scRNA-seq papers, with or without TCR/BCR data, in relation to neurological diseases of autoimmune, neurodegenerative, infectious, or oncogenic origins. We present several tables summarizing the published papers on CSF single-cell transcriptomics, classified by various neurological pathologies (**Tables 1**–**5**). These tables include detailed information on the subjects involved in each study, the annotated cell types and subtypes, and whether single-cell lymphocyte receptors were assessed. The included studies are highly heterogeneous, with some involving very few samples or lacking appropriate control groups. Some of these papers include original samples, while others reanalyze previously published datasets or integrate their own datasets with publicly available ones. Certain studies focus on specific cellular subtypes or FACS-sorted cells. Additionally, the resolution of cell type annotations, particularly for CD4+ and CD8+ T cells, is sometimes inadequate for drawing biologically meaningful conclusions. The annotations of different clusters vary significantly across the reviewed studies, and there is still no consensus on the various cell subtypes present in human CSF. We will discuss other limitations and challenges in the final section of the review.

**Table 1:** Multiple sclerosis.

Reference	Subjects included	Annotated cell types	TCR/BCR
*Beltrán et al., 2019* (60)	4 clinically discordant twin pairs RRMS, SCNI, SPMS, 4 co-twins SCNI, 2 encephalitis compared to 2 IIH	NKs, DCs, pDCs, monocytes, CD4+ T cells, CD8+ TRM, B cells, plasmablasts	TCR/BCR
*Ramesh et al., 2020* (50)	16 RRMS, 2 CIS vs 3 OND (Neurosarcoidosis, Atypical Neuroinflammatory Syndrome with uveitis and AQP4+ Neuromyelitis Optica) and 3 HCs (only 12 RRMS, 1 OND and 3 HCs were used for scRNA-seq)	NKs, DCs, pDCs, CD14+ monocytes, CD16+ monocytes, macrophages, CD4+ T cells, CD8+ T cells, naïve B cells, memory B cells, plasmablasts/plasma cells, megakaryocytes	scIg-seq
*Schafflick et al., 2020* (49)	6 MS vs 6 IIH	NKs CD56bright, NKs CD56dim, 2 clusters of mDCs, pDCs, classical/non-classical monocytes, granulocytes, CD4+ T cells, Tregs, Tfh, activated/non-activated CD8+ T cells, γδ T cells, naïve B cells, activated B cells, plasmablasts	
*Pappalardo et al., 2020* (48)	5 RRMS and 6 HC	DCs, pDCs, CD14+ monocytes, 3 clusters of CD4+ memory T cells, CD8+ memory T cells	TCR
*Esaulova et al., 2020* (56)	2 RRMS, 1 anti-MOG disorder, 2 HIV (Farhadian et al. (121))	NKs, cDCs, pDCs, CD14+ monocytes, microglía, 2 clusters of CD4+ T cells, naïve CD4+ T cells, 3 clusters of CD8+T cells, naïve CD8+ T cells, γδ T cells, 2 clusters of B cells	
*Hiltensperger et al., 2021* (46)	6 RRMS	CD4+ T cells, T regs, Th1, Th2, Th17	TCR
*Straeten et al., 2022* (47)	20 RRMS (4 from Schafflick et al. (49), 11 from Ramesh et al. (50), 3 from Heming et al. (118)) vs 2 HCs (Ramesh et al., 2021) and 9 IIH (4 from Schafflick et al. (49), 5 from Heming et al. (118))	NKs, NKs CD56+, cDCs, pDCs, CD14+ monocytes, CD16+ mono, **i**nnate lymphoid cells, cytotoxic CD4+ T cells, CD4+ naïve T cells, CD4+ proliferating, CD4+ TCM, CD4+ TEM, CD8+ naïve T cells, CD8+ proliferating, CD8+ TEM, CD8+ TCM, MAIT, Tregs, CD4- CD8- T cells, γδ T cells, naïve/ intermediate/ memory B cells, plasmablasts	
*Ostkamp et al., 2022* (45)	29 RRMS of which 5 treated with Natalizumab (2 from Esaulova et al. (56), 4 from Schafflick et al. (49), 5 from Heming et al. (118), 12 from Ramesh et al. (50) vs 9 IIH (4 from Schafflick et al. (49), 5 from Heming et al. (118)) and 3 HCs (Ramesh et al. (50)	NKbright, NKdim, tissue-resident NKs, **i**nnate lymphoid cells, AXL+ SIGLEC6+ mDCs, CD1C+ mDCs, CLEC9A+ mDCs, monocytes, MRC1+ BAMs, EMP3+ BAMs, CCL2+ microglia, CX3CR1+ microglia, TREMhigh microglía, CD4+ TEMRA, Tregs, Th1, Th2/Th22, Th17, CCR5high Th17.1, Tfh, CD8+ TCM, HLA-DR+ CD8+ TEM, CD160+ CD8 TEM, ITGA1- CD8+ TCM, ITGA+ CD8+ TRM, CD8+ CTL, V2- γδ T cells, V2+γδ T cells, MAIT, CD4+ TEMRA, B cells	
*Lindeman et al., 2022* (54)	21 MS (20 RRMS and 1 SPMS)	antibody-secreting cells, naïve B cells, memory B cells	BCR
*Seyedsadr et al., 2023* (58)	IL-11R+ cells from 2 untreated RRMS patients	NKs, mDCs, pDCs, intermediate/classical/non-classical monocytes, neutrophils, basophils, megakaryocytes, naïve CD4+ T cells, CD4+ TEM, Tregs, Th1, Th1/Th17, Th17, Th2, CD8+ TCM, CD8+ TEM, naïve CD8+ T cells, CD8+ EM; Vd2+ γδ T cells, Vd2- γδ T cells, MAIT, switched memory B cells, exhausted Bc, non-switched memory B cells, plasmablasts	
*Shevtsov et al., 2023* (62)	6 MS vs 6 IIH (Schafflick. et al. (49))	NKs, 2 clusters of mDCs, GZM B+ pDCs, monocytes, granulocytes, αβ T cells, γδ T cells, activated/ non-activated CD8+ T cells, naïve B cells, plasma cells	
*Kang et al., 2023* (53)	6 Ab-IDD, 9 MS patients (6 from Schafflick, D. et al. (49)) vs 6 IIH (Schafflick, D. et al. (49)) and 2 without inflammatory disease in CNS (1 with peripheral neuropathy and 1 with degenerative neurological disease)	CD16+ NKs, XCL1+ NKs, innate lymphoid cells, cDCs, AXL+ SIGLEC6+ DCs, ACY3+ DCs, pDCs, CD14+ monocytes, microglia, neutrophils, megakaryocytes, CD4+ naïve T cells, CD4+ memory T cells, Tregs ,Th1, Th2, Th17, Tfh, CD8+ naïve T cells, CD8+ memory T cells, TRM T cells, TEM T cells, γδ T cells, MAIT, naïve B cells, memory B cells, plasma cells	
*Ban et al., 2024* (51)	33 MS vs 7 OIND (Chronic inflammatory demyelinating polyneuropathy, Neurosarcoidosis and Transverse mielitis), 33 NIND (Cerebrovascular disease, Functional neurological disorder, Low-grade glioma, Migraine, Myelopathy, Neuropathy, Seizure, Trigeminal neuralgia) and 8 IIH	NKs, innate lymphoid cells, cDCs, AXL+ cDCs, ACY3+ cDCs, pDCs, monocytes, macrophages, SPP1+ macrophages, MALAT1low macrophages, CD4+ T cells, T regs, CD8+ T cells; CD8 γδ T cells, B cells, activated B cells, plasma cells, plasmablasts	
*Kavaka et al., 2024* (61)	4 twin pairs (4 MS of which 3 RRMS and 1 SPMS, 2 SCNI vs 2 HC) and 12 RRMS vs 5 IIH as validation cohort	effector T cells, activated T cells, MAIT, NK-like T cells, T cells with naïve - into effector-associated expression patterns	TCR
*Jacobs et al., 2025* (52)	126 MS (of which 115 RRMS and 1 SPMS), 19 OIND (acute encephalitis, aseptic meningitis, basal meningitis, chronic meningoencephalitis, chronic inflammatory demyelinating polyneuropathy, CIS, CNS inflammation, CNS vasculitis, HIV-associated meningoencephalitis, Lebers Hereditary Optic Neuropathy, Lymphocytic meningitis SLE-associated, Neurosarcoidosis, NMDA encephalitis), 23 ID (acute meningoencephalitis, meningitis, neuroborreliosis, tick-borne encephalitis, VZV-associated facial neuropathy) vs 40 NIND (isolated cranial nerve palsy, diabetic thoracic radiculopathy, functional neurological disorder, hereditary spastic paraparesis, IIH, motor neuron disease, motor neuropathy, normal pressure idrocephalus, optic disc swelling, peripheral neuropathy, primary headache)	CD16+ NKs, mDCs, pDCs, classical/non-classical monocytes, CD14+ monocytes, CD16+ monocytes, macrophages, pDCs, mDCs, TCM/naïve Th; TEM/TRM cytotoxic T cells; TEM/TEMRA cytotoxic T cells , Th1, Tregs, Tfh, memory CD4+ cytotoxic T cells, TEM/Effector CD4+ T cells, naïve CD4+/CD8+ T cells, MAIT, naïve B cells, memory B cells, plasma cells	TCR/BCR
*Rodriguez et al., 2025* (57)	5 RRMS	NKs, classical/non-classical monocytes, CD206high CD209high monocytes, cDCs, pDCs, CD4+ T cells, CD8+ T cells, MAIT, B cells	

Sequencing platforms used: 10x for all the papers, with the exception of *Beltrán et al.*, *Lindeman et al.*, and *Kavaka et al.* that use Smart-Seq2.

**Table 2:** Other autoimmune neurological diseases.

Pathology	Subjects included	Annotated cell types	TCR/BCR
Neuromyelitis optica			
*Zhang et al., 2021* (64)	4 NMOSD	naïve B cells, memory B cells, age-associated B cells, MS4A1high antibody-secreting cells, CD27high antibody-secreting cells, MK67high antibody-secreting cells, SDC1high antibody-secreting cells	BCR
*Qin et al., 2024* (65)	6 NMOSD vs 2 IIH and 1 with primary headache	NKs, cDCs, pDCs, CD14+ monocytes, CD16+ monocytes, macrophages-like cells, microglia-like cells, CD4+ T cells, CD8+ T cells, B cells, plasma cells	
*Qin et al., 2024* (18)	5 NMOSD vs 5 HC	NKs, cDCs, pDCS, CD14+ monocytes, CD16+ monocytes, macrophages, CD4+ T cells, T regs, CD4+ TCM, CD4+ TEM, cytotoxic CD4+ T cell, CD8+ T cells, CD8+ TCM, CD8+ TEM, CD8+ TE, NK-like T cells, cycling T cells, naïve B cells, memory B cells (switched/non-switched), double-negative B cells, immature B cells	TCR/BCR
Antibody-mediated encephalitis			
*Li et al., 2024* (69)	3 NMDAR-E vs 6 IIH (Schafflick, D. et al. (49)) and 5 VE (Heming, M. et al. (118))	DCs, pDCs, monocytes/macrophages, megakaryocytes, CD4+ T cells, CD8+ T cells, γδ T cells, naïve B cells, classical memory B cells, IgM memory B cells, plasma cells	BCR
*Theorell et al., 2024* (68)	2 LGI1 + 1 CASPR2 antibody encephalitis	T and B cells sorted by flow cytometry	TCR/BCR
Neurosarcoidosis			
*Paley et al., 2022* (71)	5 with probable Neurosarcoidosis vs 1 PD and 1 with BSCR	NKs, myeloid cells, naïve T cells, CD4+ T cells, 10 clusters of CD8+ T cells, B cells	TCR/BCR

Sequencing platforms used: 10x for all the papers, with the exception of *Theorell et al.* that use Smart-Seq2.

**Table 3:** Neurodegenerative diseases and narcolepsy.

Pathology	Subjects included	Annotated cells types	TCR/BCR
Alzheimer’s disease			
*Gate et al., 2020* (77)	7 AD, 5 MCI vs 14 HCs; 8 PD	NKs and other innate cells, 6 clusters of CD4+ T cells, CD4+ CD8+ T cells, CD8+ TEM, CD8+ TEMRA, plasma cells	TCR
*Piehl et al.,2022* (17)	6 AD, 8 MCI vs 45 HCs	NKs, DCs, classical/ non-classical/ intermediate monocytes, CD4+ T cells, CD8+ T cells, CD4+ CD8+ T cells, T regs, B cells, plasma cells	TCR
*Wang et al.,2024* (89)	4 AD, 5 MCI vs 9 HCs (Gate et al. (77))	NKs, myeloid cells, 3 clusters of CD4+ T cells, naïve CD4+ T cells, CD4+ TCM, CD4+ TEM, CD8+ TEM, CD8+ TEMRA, B cells	TCR
Parkinson’s disease			
*Gate et al., 2021* (19)	9 PDD, 2 DLB vs 11 HCs	NKs, DCs, monocytes, CD4+ T cells, CD8+ TEM, GNLY+ CD8+ T cells, CD4- CD8- T cells, B cells, plasma cells	TCR
*Wang et al., 2021* (106)	6 PD vs 9 HCs (Gate et al. (77))	naïve CD4+ T cells, CD4+ TCM, CD4+ cytotoxic T cells, Th1, Th2, Th17, Tfh, T regs, naïve CD8+ T cells, CD8+ TCM, transitional CD8+ T cells, CD8+ TEM, MAIT, γδ T cells, CD4- CD8- T cells	TCR
*Guan et al., 2022* (105)	7 PD vs 7 HCs (Gate et al. (19))	NKs, DCs, pDCs, monocytes, CD4+ T cells, naïve CD4+ T cells, CD8+ T cells, cycling T cells, B cells	
Amyotrophic lateral sclerosis			
*Yazdani et al., 2022* (110)	5 ALS vs 2 normal pressure hydrocephalus,1 cervical radiculopathy and 1 HC	NKs, 2 clusters of cDCs, CD16+ DCs, pDCs, CD16- monocytes, CD16+ monocytes, megakaryocytes, activated/non-activated CD4+ T cells, cytotoxic CD4+ , T regs, activated/non-activated CD8+ T cells, γδ T cells, CD4+ CD8+ T cells, B cells	TCR
Spinal muscular atrophy			
*Lu et al., 2024* (112)	9 SMA vs 9 IIH	NKs, DCs, pDCs, monocytes, macrophages, granulocytes, naïve CD4+ T cells, effector CD4+ T cells, memory CD4+ T cells, CD4+ NK T cells, cytotoxic CD8+ T cells, B cells	TCR
Narcolepsy			
*Huth et al., 2024* (115)	14 Narcolepsy vs 3 MS, 2 RIS and 5 IIH	NKs, DCs, pDCs, monocytes, CD4+ T cells, Tregs, naïve T cells, Th1 TCM, Th1 TEM, Th17, naïve CD8+ T cells ,CD8+ TCM, CD8+ TEM, CD8+ TRM, MAIT, KIR+ T cells, γδ T cells, B cells, plasmablasts	TCR

Sequencing platforms used: 10x for all the papers.

**Table 4:** Neurological infectious diseases.

Pathology	Subjects included	Annotated cells types	TCR/BCR
Neuro-COVID			
*Heming et al., 2021* (118)	8 NeuroCOVID vs 9 IIH, 9 MS (Schafflick, D. et al. (49)) and 5 VE	NK, mDCs, mature DCs, pDCs, monocytes, granulocytes, CD4 T cells, naïve CD4+ T cells, CD4+ proliferating, memory-like T cells, exhausted CD4+T cells, antiviral CD4+ T cells, T regs, CD8+ T cells, γδT cells, naïve B cells, plasma cells	TCR
*Song et al., 2021* (119)	5 NeuroCOVID vs 11 HC (8 from Gate et al. (77))	NKs, DCs, classical/ non-classical/ intermediate monocytes, CD69+ CD4+ T cells, IL7R+ CD4+ T cells, Th1, Th2, Th17, naïve CD4+ T cells, T regs, CD4+ CD8+ T cells, CD4- CD8- T cells, KLRG1+ CD8+ T cells, MPEC CD8+ T cells, naïve CD8+ T cells, B cells, dark zone-like B cells, CD86high B cells, immature B cells, memory B cells, IgG plasma cells, IgM+ cells, plasma cells, IgA+ cells	BCR
Neurological HIV			
*Farhadian et al., 2018* (121)	3 HIV on antiretroviral therapy vs 2 non-infected	NKs, microglia-like cells, 4 clusters of T cells, CD8+ T cells, B cells, plasma cells	
*Pinnetti et al., 2024* (124)	1 MS HIV+	NKs, mDCs, pDCs, monocytes, granulocytes, megakaryocytes, CD4+ T cells, CD8+ T cells, B cells, plasma cells	
Progressive multifocal leukoencephalopathy			
*Deffner et al., 2024* (126)	3 O-PML (1 from Touil et al. (157)) vs 9 IIH (4 from Schafflick et al. (49) and 9 from Heming et al. (118)), 12 RRMS (5 from Schafflick et al. (49), 5 from Heming et al. (118), 3 from Touil et al. (157), 5 RRMS-Nat (Ostkamp et al. (45)), 5 VE (Heming et al. (118))	NKs bright, NKs dim, innate lymphoid cells, monocytes, CD1C+ mDCs, AXL+SIGLEC6+ mDCs, CLEC9A+ mDCs, pDCs, MRC1+ BAMs, EMP3+ BAMs, CCL2+ microglia, CX3CR1+ microglia, TREM2high microglía, CD4+ TEMRA, Tregs, Th1, Th17, CCR5high Th17.1, Th2/Th22, Tfh, CD8+ TCM, CD8+ CTL, CD8+ EM T cells, CD8+ EM CD160+ T cells, CD8+ EM HLA-DRA+ T cells, CD8+ TRM ITGA1-, CD8 TRM ITGA1+, γδ T cells Vd2-/+ T cells, MAIT, B cells activated, B cells atypical, IL4R+ B cells, plasmacells	
Bacterial meningitis			
*Xiao et al., 2022* (127)	33 patients at different stages of BM	NKs, DCs, pDCs, monocytes, macrophages, granulocytes, naïve CD4+ T cells, effector CD4+ T cells, memory CD4+ T cells, CD4+ NK T cells, cytotoxic CD8+ T cells, B cells	
Tuberculous meningitis			
*Mo et al., 2024* (129)	6 TBM, 1 VM and 1 CM	NKs, cDCs, pDCs, classical/non-classical monocytes, macrophages, microglia, neutrophils, naïve CD4+ T cells, CD4+ TCM, CD4+ TRM, Th1, Th17, T regs, CD4+ exhausted T cells, naïve CD8+ T cells, CD8+ TEM, CD8+ TRM, MAIT, naïve B cells, memory B cells, plasma cells, plasmablasts	
Subarachnoid neurocysticercosis			
*Tang et al., 2022* (132)	5 SANCC vs 7 controls (Touil et al. (157)	mDCs, pDCs, CD4+ T cells, CD8+ T cells, FoxP3+ natural T regs, FoxP3- induced Tregs, naïve B cells, antibody-secreting cells	

Sequencing platforms used: 10x for all the papers, with the exception of *Farhadian et al.* that use SeqWell and *Xiao et al.* that use QUARTZ-SEQ2.

**Table 5:** Brain cancer and metastasis.

Pathology	Subjects included	Annotated cells types	TCR/BCR
Glioblastoma			
*Wang et al., 2024* (133)	1 patient with GBM and 1 with non-malignant brain tumor	NKs, DCs, monocytes, macrophages/microglia, astrocytes, T cells, B cells, plasma cells	
Brain and leptomeningeal metastasis			
*Ruan et al., 2020* (137)	5 with LUAD LMs, 1 CUP LMs vs 3 normal controls from patients who had pulmonary cryptococcal infection without CNS symptoms	monocytes, T cells, B cells, circulating tumor cells	
*Chi et al., 2020* (138)	5 with LMs (3 from BC, 2 from LC)	NKs, cDCs, pDCs, 2 clusters of monocytes, macrophages, CD4+ T cells, CD8+ T cells, B cells, 5 clusters of tumoral cells	
*Rubio-Perez et al., 2020* (20)	3 LUAD with BrM, 1 ESCA with BrM, 1 BRCA with BrM, 1 SKCM with BrM	NKs, DCs, 2 clusters of TAMs, microglia, neutrophils, cytotoxic T cells, naïve T cells, T regs, 2 clusters of B cells, CAFs	TCR
*Prakadan et al., 2021* (140)	19 with LMs of which 10 under PD-1 inhibitor treatment and 9 under PD-1 and CTLA4 inhibitor treatment	NKs, DCs, pDCs, monocytes, macrophages, T cells, immunoglobulin-expressing B cells, tumor cells	
*Li et al., 2021* (67)	4 with LMs from NSCLC	NSCLC LM cells	
*Smalley et al., 2021* (141)	6 with LMMs vs 2 with no LMMs	NKs, pDCs, cDCs, monocytes-derived DCs, monocytes, monocytic myeloid derived suppressors, macrophages, activated CD4+ T cells, exhausted CD4+ T cells, Tregs, CD8+ T cells, CD8+ TEM, CD8+ TCM, B cells, activated B cells, plasma cells, memory B cells, marginal zone B cells, follicular B cells, regulatory B cells, melanoma cells	
*Ruan et al., 2022* (136)	4 with BC-LM (3 from Chi et al. (138)), 2 LC-LM (Chi et al. (138)) vs 4 IIH (Schafflick et al. (49))	NKs, 2 clusters of mDCs, pDCs, monocytes, TAMs, conventional macrophages, naïve CD4+ T cells, T regs, cytotoxic CD8+ T cells, CD8+ exhausted T cells, B cells, 5 subtypes of circulating tumor cells	
*Zhou et al., 2024* (135)	12 with BrMs from BC	cDCs, pDCs, mononuclear macrophages, TAMs, cytotoxic T cells, naïve T cells, CD8+ T cells, CD4+ T cells, T regs, endothelial cells and fibroblasts, glioblastals	
*Li et al., 2024* (139)	4 with BrMs and 6 with LMs from NSCLC	DCs, monocytes, perivascular macrophages, CNS BAMs, microglia, T cells, B cells, plasma cells, epithelial cells	
Central nervous system lymphoma			
*Ruan et al., 2021* (130)	5 PCNSL-DLBCL, 2 SCNSL-DLBCL vs 3 normal controls (Ruan et al. (137))	monocytes, T cells, B cells, diffuse large B cells, dark zone B cells, intermediate B cells, light zone B cells, plasmablasts, precursor memory B cells, diffuse large B cell	
*Ruan et al., 2022* (145)	1 PL-ALCL vs 3 normal controls (Ruan et al. (137))	monocytes, T cells, B cells, anaplastic large cells	
*Heming et al., 2022* (144)	1 PCNSL	NKs, mDCs, monocytes, granulocytes, olygodendrocytes, naïve/memory-like CD4+ T cells, T regs, naïve/memory-like CD8+ T cells, proliferating T cells, exhausted T cells, 4 clusters of malignant B cells, 2 clusters of non-malignant B cells	BCR

Sequencing platforms used: Smart-Seq2 for Ruan et al. (137), Ruan et al. (130), Ruan et al. (145); SeqWell for Prakadan et al. (140); Fluidigm C1.

for *Li et al.* (67); Singleron for *Zhou et al.* (135); BD Rhapsody for *Li et al.* (139); 10x for all the remaining papers.

In the specific cases of multiple sclerosis (MS), Alzheimer’s disease (AD), and Parkinson’s disease (PD), we will also analyze the results obtained from studies using postmortem brain tissue regarding brain-infiltrating cells, both at the parenchymal and perivascular levels. We will also briefly contextualize the findings obtained with single-cell transcriptomics in the CSF with those obtained using other cytometry technologies. In principle, the results obtained from the CSF should be consistent with the findings observed in the brain.

Among the various published scRNA-seq studies, we highlight a recently published article that includes a substantial number of controls and covers many neurological diseases (22). Although it does not include the analysis of lymphocyte receptors, it achieves a high level of resolution in the annotations of different immune cell subtypes, allowing for relevant conclusions. Cantoni and collaborators integrate different published datasets, and the dataset generated with their own samples. Samples were divided into five groups: healthy controls, multiple sclerosis, neurodegenerative diseases, infectious central nervous system (CNS) diseases, and other autoimmune diseases. A general key discovery was the identification of three microglia-like subclusters in the CSF-CCL2+ microglia, SPP1+ microglia, and FN1+ microglia. Among these, FN1+ microglia cells were found to be particularly elevated in neurodegenerative diseases, suggesting their potential role in disease progression or response. Another significant finding was the presence of a previously unreported dendritic cell (DC) subset, AREG+ cDC2, which was exclusive to the CSF and exhibited increased frequency in MS patients, pointing to a potential role in MS pathology. The findings for each type of pathology will be discussed in detail in their respective sections and are summarized in **Table 6**.

**Table 6:** Integrated analysis of different neurological diseases (*Cantoni et al.* (22)).

Cohort	CD4+ T cells	CD8+ T cells	B-cell Lineage	Innate Immune cells
Multiple Sclerosis	Increase in Tfh cells; decrease in Th1 cells.	Higher frequency of naive CD8+ T cells; absence of TEM GZMB+ cells.	Dramatic expansion of atypical memory B cells and plasmablasts; presence of CD11c+ age-associated B cells.	Reduction in CD16+ monocytes and microglia-like cells; increase in AREG+ cDC2; altered NKs composition with a decrease in cytotoxic NKs (CD56dimLAG3–) and an increase in tissue-resident NKs (CXCR6+ ITGAE+).
Neurodegenerative Diseases	Increase in Th1 cells; no significant elevation of Tfh cells.	Enrichment of CD8+ TEM GZMB+ cells.	Mild increase in plasmablasts in some patients with Alzheimer's disease and dementia with Lewy bodies.	Increase in FN1+ and SPP1+ microglia-like cells; no elevation of CD16+ monocytes.
Infectious CNS Diseases	Strong Th1 response; increase in Th Ifn cells.	Significant reduction in CD8+ HLA-DR+ T cells.	Striking expansion of plasmablasts.	Strong increase in CD16+ monocytes, LAMP3+ DCs, and neutrophils.
Other Inflammatory Diseases	Strong increase in Tfh cells in autoimmune encephalitis and neuromyelitis optica spectrum disorder.	No significant expansion of CD8+ T cells.	High expansion of B cells, particularly in neuromyelitis optica spectrum disorder and autoimmune encephalitis; significant abundance of plasmablasts.	Increase in DCs and monocytes in autoimmune encephalitis; no alteration in microglia-like cells.

Sequencing platform used: 10x.

## 2 Multiple sclerosis

MS is a chronic autoimmune disease characterized by the demyelination of nerve fibers in the CNS. The disease can manifest in various clinical forms, including relapsing-remitting (RRMS) and progressive types. Clinically isolated syndrome and radiologically isolated syndrome are considered early stages of MS. Four distinct lesion patterns have been identified in the early stages of MS, suggesting that MS may be a pathologically heterogeneous syndrome (4). Identifying the triggers involved in the diverse pathological processes and developing more targeted treatments with fewer adverse effects is crucial. Historically, MS has been considered primarily a T cell-driven disease. CD4+ T helper cells have long been considered the primary culprits in the immunopathogenesis of MS, largely due to the strong association with specific MHC class II alleles and their role in inducing experimental autoimmune encephalomyelitis (5). However, there is increasing evidence pointing to the importance of CD8+ T cells in the onset of the disease (5–8). The involvement of B cells in MS is inferred from the presence of oligoclonal bands in the CSF of around 90% of patients (9), their clonal expansion in lesions, the deposition of antibodies and complement in a significant portion of patients (10), and most notably, the effectiveness of treatments aimed at their depletion (11). However, its precise role in the etiopathogenesis of the disease is not clear. Although B cells are believed to play a role in the pathology of MS by producing autoantibodies that target CNS antigens, it is now understood that B cells play a role in MS relapses through mechanisms that do not involve antibodies (12). These include presenting antigens to T cells and releasing pro-inflammatory cytokines. Additionally, the discovery of B cell-rich follicle-like structures in the meninges of patients with progressive MS indicates that B cells also contribute to the disease’s progression during its later stages (13).

### 2.1 Brain-infiltrating immune cells in multiple sclerosis

#### 2.1.1 Presence of B-cell lineage cells in the perivascular space, intrameningeal follicles, and parenchyma

In MS brains, B cells are concentrated in the perivascular space of one or a few larger veins within the plaque centers and are rarely found around small vessels or diffusely in the lesion parenchyma. The number of B cells in lesions varies greatly, ranging from none in some progressive MS patients to four times the number of T cells in others (23). As mentioned before, proliferating B cells are found in intrameningeal follicles with a predominance of germinal-center-like B cell clusters (24), while nonproliferating plasma cells (CD138+Ki67−) are present in brain tissue (25). Related B cell clones are found in both the meninges and parenchyma of patients with MS (26).

#### 2.1.2 CD8+ T cells predominate in multiple sclerosis lesion

Lymphocytic perivascular cuffs are most prominent at the periphery of active plaques and are occasionally observed in regions without evidence of demyelination or macrophage infiltration. These cuffs predominantly comprise T cells and MHC-II+ cells, with a high presence of CD8+ cells and variable numbers of CD4+ cells. The CD8/CD4 ratios within the cuffs range from 1:1 to 50:1. In normal-appearing white matter, cuffs are sparse and mainly consisted of CD8+ T cells. The distribution of T cells in the parenchyma mirrors that in the perivascular cuffs, being predominantly CD8+ cells with variable numbers of CD4+ cells (27). Despite CD4+ T cells generally being outnumbered by CD8+ T cells in brain lesions (23, 27–30), their role in triggering local pathology in MS cannot be ruled out. A significant presence of CD4+ T cells in pre-active lesion sites suggests their involvement in the early stages of lesion formation (28). However, MS donors with a low CD8/CD4 ratio in different brain lesions exhibit a higher proportion of inactive remyelinated areas compared to those with a high CD8/CD4 ratio (29).

Parenchymal infiltration is observed in active and mixed active/inactive lesions, but not in inactive lesions (29). Further characterization revealed that these cells are enriched for CD20^dim^ tissue-resident memory (TRM) CD8 T cells, which are often CD103-negative (30). These TRM CD8 T cells are reactivated in acute, relapsing, and progressive MS lesions (23). It has been shown that TRM CD8 T cells expressing granzyme B, but lacking perforin, can still kill neurons in MS (23, 31).

#### 2.1.3 Brain-infiltrating Epstein-Barr virus-infected B cells recruit CD8+ T cells

Recent epidemiological studies have provided strong evidence that MS is an uncommon complication of infection with the Epstein-Barr virus (EBV), a herpesvirus that infects more than 90% of the population (32). Other studies have demonstrated a strong association between EBV infection and MS, with CD8+ T cells (33, 34), particularly CD8 TRM (35), playing a significant role in this process. Analysis of postmortem MS brain samples revealed the expression of the EBV lytic protein BZLF-1 and interactions between cytotoxic CD8+ T cells and EBV-infected plasma cells in inflammatory lesions, suggesting that failure to control EBV infection could lead to intracerebral viral reactivation and disease relapse. These studies indicate that persistent EBV infection in the CNS stimulates a CD8+ T-cell response aimed at clearing the virus, but this response inadvertently causes CNS injury. In MS brain tissue, CD8+ T cells recognizing EBV proteins were found in white matter lesions and meninges (36, 37). Interestingly, clonally expanded CD8+ T cells reactive to autologous EBV-infected B cells are found forming immune synapses in the brain parenchyma of MS donors (34). The presence of these expanded CD8+ T cells can be detected in the CSF even at the earliest stages of MS (38, 39), suggesting that they likely play an important role in pathogenesis. The expression of immune checkpoint molecules like PD-L1 on EBV-infected B cells and PD-1 on CD8+ T cells may facilitate local immune evasion, leading to EBV persistence and ongoing inflammation in the MS brain (40).

#### 2.1.4 Indeterminate role of innate immune cells

scRNA-seq data suggest that brain macrophages exhibit an early stress response in normal-appearing white matter, indicating they might be among the first cells involved in the onset of demyelination (41). However, the exact role of this stress response is not well understood. It could also be a protective mechanism by these cells, aiming to prevent lesion formation in the affected tissue area. Similarly, microglia in normal-appearing white matter exhibit an alert state while displaying features of immunosuppression, suggesting they are responding to ongoing neuroinflammation (42). Therefore, it is not clear whether macrophages/microglia have a deleterious role, as previously suggested. In a mouse model, it has been shown that both microglia and monocyte-derived macrophages engulf equal amounts of myelin debris following demyelination. Furthermore, these macrophages can compensate for microglia depletion by enhancing their phagocytic activity. Importantly, microglia have been demonstrated to promote the recruitment of oligodendrocyte progenitor cells, facilitating their differentiation and subsequent remyelination (43).

Single-cell mass cytometry profiling revealed a periventricular accumulation of both CD8+ and CD4+ T cells as well as CD56^bright^ natural killer (NK) cells in multiple sclerosis. CD56^bright^ NK cells expressed higher levels of proteins involved in NK cell activation and migration suggesting a role in MS (44).

### 2.2 CSF single-cell transcriptomics in multiple sclerosis

All articles related to multiple sclerosis are compiled in **Table 1**.

#### 2.2.1 Peripheral and brain origin of CSF immune cells

The origin of immune cells present in the CSF can be either peripheral or derived from nervous tissue. A study investigated whether human CSF contains TRM cells and CNS-resident myeloid cells from the parenchyma or border tissues (45). By comparing CSF immune cells from multiple sclerosis relapse with those collected during therapeutic very late antigen-4 blockade, researchers identified immune subsets from various lineages, including CNS border-associated macrophages, CD8 and CD4 TRM cells, and tissue-resident NK cells. All lymphocytic CNS-resident cells expressed *CXCR6* but differed in ITGAE expression (encoding CD103). CD4 and CD8 TRM cells shared a common signature with *ZFP36L2*, *DUSP1*, and *ID2* expression. The researchers also developed a user-friendly application to facilitate the transfer of cell identities from a reference atlas to new CSF scRNA-seq data (45).

In an animal model of MS, T cells from different peripheral sites exhibit distinct signatures based on their origin. Site-specific labeling revealed unique characteristics of inguinal and mesenteric T cells in the inflamed CNS, though these signatures were not clearly extrapolated to CD4+ subsets in MS CSF cells (46).

#### 2.2.2 CSF cellular composition in multiple sclerosis

The most consistent findings regarding cell type composition among various scRNA-seq studies on the CSF of MS patients is the increase in antibody-secreting cells (ASC) (plasmablasts and plasma cells) and memory B cells (22, 45, 47–52), the expansion of B-cell-helping T follicular helper (Tfh) cells (22, 45, 48, 49, 52, 53), increased proportions of regulatory T cells (Treg) (45, 48, 49), and higher numbers of NK cells (22, 45, 47, 49).

B and plasma cells are found to be enriched in MS patients who are oligoclonal band-positive and those with active disease (51). A study integrating multiple scRNA-seq datasets of CSF cells from patients with early RRMS demonstrates that the expanded CSF B lineage cells resembled class-switched ASC (47). Interestingly, a study that performed single-cell full-length RNA-seq and BCR reconstruction in the CSF of MS patients demonstrated an association between IgG constant region polymorphisms and stereotyped B-cell responses in MS. This suggests that the intrathecal B-cell response in these patients may target structurally similar epitopes (54). Regarding NK, an increase in absolute NK cell counts has been reported in flow cytometry settings and is associated with MS activity. MS patients showed a significant increase in the regulatory/effector (CD56^bright^/CD56^dim^) NK cell ratio compared to other inflammatory neurological diseases (OIND) and non-inflammatory neurological diseases (NIND) groups, suggesting that regulatory NK cells are attempting to counteract adaptive immune activation (55). An increase in both CD56^bright^ and CD56^dim^ NK cells was observed in MS after scRNAseq analysis (49), but the exact role of the different NK cell subsets still needs to be clarified.

A study including two patients with RRMS and one with anti-MOG disorder, identified cells exhibiting a transcriptomic signature akin to microglia. These microglia-like cells were distinguishable from other myeloid cell populations through flow cytometry. The presence of microglia within the human CSF, detectable by surface protein expression, underscores their potential role in neuroinflammation (56). However, these microglia-like cells are also found in non-inflammatory conditions (22).

Cantoni and collaborators study included subjects with RRMS and clinically isolated syndrome, highlighting MS as a highly compartmentalized immune disorder characterized by intrathecal B cell expansion, altered T cell dynamics, and innate immune involvement (**Table 6**) (22). MS patients exhibited a significant increase in Tfh cells in the CSF, supporting B cell activation and antibody production, along with a decrease in Th1 cells in the CSF and a marked increase in Th17 cells in the blood. Additionally, there was a higher frequency of naive CD8+ T cells in the CSF, suggesting recruitment from the periphery, while effector memory CD8+ GZMB+ T cells were absent, indicating a different mechanism of immune activation compared to neurodegenerative diseases. MS patients also showed a reduction in CD16+ monocytes (non-classical) and microglia-like cells, distinguishing MS from other inflammatory conditions, but had significantly increased AREG+ cDC2 DC in the CSF, supporting their role in disease progression. NK cell composition was altered, with a decrease in cytotoxic NK cells (CD56d^im^LAG3–) and an increase in tissue-resident NK cells (*CXCR6*+ *ITGAE*+), suggesting selective retention within the CNS. Furthermore, MS was marked by a dramatic expansion of atypical memory B cells and plasmablasts in the CSF, with CD11c+ age-associated B cells indicating a chronic inflammatory state, reinforcing the dominant role of B cells in MS pathogenesis.

An increase of DC has also been reported by other single-cell studies (49). One of the studies revealed that AXL+SIGLEC6+ DC (ASDCs) are particularly overrepresented in the CSF of patients with inflammatory demyelinating disease, including antibody-mediated disease and MS, compared to controls (53). ASDCs were also found in brain tissues during acute attacks, often in close contact with T cells, suggesting a role in CNS autoimmunity. However, given that the controls consist of samples from only one peripheral neuropathy and one degenerative neurological disease, it is advisable to confirm the results with more appropriate and comprehensive controls to ensure their validity. Another study reported enrichment with a rare subtype of CD8+ T cells in MS compared to other pathologies (51). This subtype is characterized by the expression of cytotoxic markers (*GZMA*, *GZMK*), upregulation of the co-stimulatory marker CD27, upregulation of inhibitory receptors (*HAVCR2*, *TIGIT*), and downregulation of chemokines (*CCL4*, *CCL5*). Regarding monocytes, a recent mass cytometry and scRNA-seq combined analysis demonstrated an enrichment of a classical monocyte population in 22% of MS patients at the time of diagnosis, that predicted a more aggressive progression of the disease (57). This monocyte population is found more frequently in MS patients carrying the HLA-DRB1*15:01 allele both in blood and CSF. It is characterized by *CD206*, *CD209*, *CCR5* and *CCR2* expression and exhibited antigen-presenting abilities along with a pro-inflammatory profile. In addition, another study involving blood and CSF scRNAseq analyses with sorted cells suggests that IL-11 induces NLRP3 inflammasome activation in monocytes and promotes the migration of inflammatory cells to the central nervous system (58).

#### 2.2.3 Comparison of multiple sclerosis to other inflammatory and infectious diseases

Cantoni and collaborators identified several similarities between MS and other neuroinflammatory diseases, such as a strong increase in Tfh cells, B cells, and plasmablasts (**Table 6**). However, the other neuroinflammatory diseases included in the study did not exhibit an expansion of CD8+ T cells, AREG+ cDC2 DC, or a reduction in Th1 cells, CD16+ monocytes, and microglia-like cells. Nonetheless, an overall increase in DC was observed (22). On the other hand, they did not find an increase in Tfh cells in infectious diseases or other alterations observed in MS patients, except for the expansion of plasmablasts. However, they did identify certain exclusive changes in the group of infectious diseases. These results contrast with those reported in another recently published study, although the diseases included in neuroinflammatory, and infectious diseases groups were not the same. This study that integrates single-cell gene expression with lymphocyte receptor sequencing concludes that most of the changes observed in MS are common to other neuroinflammatory diseases (52). Jacobs and collaborators included in the study the largest number of CSF MS samples to date, with 123 cases of untreated MS, 19 patients with OIND, 23 patients with infectious neurological diseases (IDs), and 36 patients with NIND. However, the study did not include a healthy control group and instead used NIND as the reference group. The study shows that clonal expansion of CSF B and T cells is observed across different neurological diseases but is most prominent in inflammatory diseases such as MS. It is important to note that no MS-specific cell subsets were identified in this study. They found that neuroinflammatory diseases, including MS, exhibit an enrichment of most of the cell subsets reported by other studies (52). For example, the rise in Tfh cells was noted across all three inflammatory groups (MS, OIND, and ID), but not in the non-inflammatory controls. They suggest that the presence of these cells in CSF might be characteristic of neuroinflammatory CNS disorders. While MS CSF cellular composition was like OIND CSF, it contained lower relative proportions of NK and Tregs compared with ID CSF. SUB1, which is involved in B cell differentiation into ASC, is the most highly expressed gene in MS and ID. The fact that, for example, the increase in ASC, B cells, and T regulatory cells is common to other neuroinflammatory diseases (51) and inflammatory demyelinating diseases (53) was reported before by other single-cell transcriptomic studies.

In the study by Jacobs and collaborators, an increase in markers of tissue residence, antigen presentation, cytotoxicity, and proliferation were observed across cell types and cohorts (52). Gene Set Enrichment Analysis (GSEA) revealed enrichment of interferon γ (IFN-γ) signaling and complement pathways in expanded clones across various cell types. The genes defining these clones were consistent across cohorts, suggesting a general transcriptional program linked to clonal expansion rather than an MS-specific occurrence. The level of clonality in the CSF T cell compartment showed no significant difference between patients with MS and those with NIND. Interestingly, it was lower in MS patients compared to those in the ID cohort. The TCR repertoire in CSF was polarized toward memory and effector T cell subsets, with clonally expanded T cells present in both inflammatory and non-inflammatory conditions. Clonally expanded cells in the CSF were predominantly of a CD8+ tissue-resident effector memory phenotype and were enriched for EBV-specific and CMV-specific CDR3 sequences in MS cases and NINDS. The authors accurately note that the clonal expansion of T cells recognizing these viral antigens, identified through bioinformatic antigen-prediction tools, may be influenced by the biased nature of public databases where these epitopes are predominant. This bias may not precisely reflect the true proportion of antigens recognized by the clonally expanded T cells.

#### 2.2.4 Expression quantitative trait loci analysis and impairment of viral control

Jacobs and collaborators conducted cis-expression quantitative trait loci (eQTL) mapping in CSF CD4+ T cells, CD8+ T cells, and B cells focusing on genes that were upregulated in CSF and have been implicated in MS pathogenesis through GWAS (52). They found substantial evidence that the MS risk allele alters the expression of several genes (*ORMDL3*, ANKRD55, *FCRL3*, *AHI1*, *EAF2*, *GDPD5*, and *ZC2HC1A*) in the CSF. They identified CSF-eQTLs, including ETS1 in CD4+ T cells and EAF2 in B cells, suggesting mechanisms for T cell migration to the CNS and B cell proliferation regulation.

A previously published study suggests that alterations in viral control mechanisms may play a crucial role in the development of MS. Altered activity in pathways responsible for controlling inflammatory and type 1 interferon responses in both T cells and myeloid cells was found in MS patients. Additionally, a systematic search for expression eQTLs highlighted two eQTLs in CD8+ T cells, fine-mapped to MS susceptibility variants in the viral control genes *ZC3HAV1* (rs10271373) and *IFITM2* (rs1059091) (51).

#### 2.2.5 Twin study reveals CD8+ T cell role in the onset and progression of MS

A very promising strategy for studying the mechanisms involved in the onset of MS is to examine identical twins. Identical twins have a 25% risk of developing the disease if their sibling has already been diagnosed with MS (59). Among individuals without MS, some exhibit no signs of brain inflammation, while others have subclinical indicators of inflammation that do not meet the criteria for an MS diagnosis. Those with subclinical indicators may be in a very early, or prodromal, stage of the disease. In these subjects, clonally expanded TRM-like CD8+ cells were found at a higher degree compared to controls, and expanded plasmablasts were observed in both MS and prodromal individuals with oligoclonal bands (60). Single-cell transcriptomic analysis of peripheral CD8+ T cells unveiled disease-associated transcriptional features that persist across both prodromal and clinically manifest stages of MS (61). These findings were substantiated by brain tissue analysis, which underscored the preservation of these immunological and metabolic signatures at sites of neuroinflammation. Using scRNA-seq and scTCR-seq analysis, T cell clonotypes with high migratory potential were found in both blood and CSF. Indeed, it has been previously demonstrated that brain-infiltrating CD8+ T cells persist as clonal expansions in both the CSF and blood (15). These clonotypes were part of T cell subsets expressing gene profiles indicative of potent effector function, activation, and a high capacity for energy production (61). Altogether, this suggests that CD8+ T cells are involved in the initiation and progression of the disease.

#### 2.2.6 Using scRNA-seq data in drug reprofiling

Publicly available data not only allows for the reanalysis of scRNA-seq data from different perspectives but has also been proposed as a tool for exploring drug repositioning. To achieve this objective, a study demonstrated that AIM2 inflammasome, SMAD2/3 signaling, and complement activation pathways are activated in MS within various CSF and peripheral blood mononuclear cells (PBMC). By leveraging genes from the most activated pathways, researchers identified several promising small molecules capable of reversing the transcriptomic signatures of MS immune cells. Notably, among these molecules, they detected the MS drug Mitoxantrone, which validates the approach (62).

## 3 Other neurological autoimmune diseases

All articles related to other neurological autoimmune disorders are compiled in **Table 2**.

### 3.1 Neuromyelitis Optica spectrum disorder and antibody-mediated encephalitis

Neuromyelitis Optica spectrum disorder (NMOSD) is a rare autoimmune condition that primarily affects the optic nerves and spinal cord (63). It is characterized by the presence of specific antibodies, such as anti-aquaporin-4 antibodies, which target and damage the CNS. Antibody-mediated encephalitis is an autoimmune condition where antibodies target brain cells, causing inflammation and neuropsychiatric symptoms. Single-cell transcriptomics of CSF, blood, and bone marrow from NMOSD patients shows that B cells are compartmentally fine-tuned toward autoreactivity in NMOSD and that B cells are hyperresponsive to type I interferon, promoting B-cell maturation and anti-aquaporin-4 autoantibody production (64). B cell lineages, particularly ASC, are expanded in the blood and CSF (18). The highly clonal CSF B cell repertoire, rich in IgG subtypes, indicated antigen-specific proliferation. Genes related to Ig production, immune responses, B cell activation, and BCR signaling are up-regulated. About 13% of the Ig VH sequences of CSF B cells in NMOSD patients match those in their blood, indicating that most CSF B cells likely originate from the CNS and not from the blood (18).

Aside from the involvement of B-lineage cells, macrophage/microglia-like cells also appear to be implicated. scRNA-seq of macrophage/microglia-like cells in CSF showed that increased CSF soluble TREM2 was positively associated with microglial dysfunction in patients with NMOSD. Supported by an animal model, the study found that excessive activation, overwhelmed phagocytosis of myelin debris, suppressed lipid metabolism, and enhanced glycolysis contribute to soluble TREM2-mediated microglial dysfunction (65).

Anti-N-methyl-D-aspartate receptor encephalitis (NMDAR-E) is a severe autoimmune disorder characterized by prominent psychiatric symptoms (66). A study assessed CSF immune cells from NMDAR-E patients using a combination of scRNA-seq, scBCR-seq, bulk BCR sequencing, flow cytometry, and ELISA (67). The CSF showed significantly increased B cell counts, predominantly memory B cells, compared to the other two groups (intracranial hypertension and viral encephalitis). CSF B cells predominantly expressed immunoglobulin heavy chain gamma, whereas B cells in peripheral blood predominantly expressed immunoglobulin heavy chain mu, suggesting that antigen class switching occurs in CSF. However, in patients with LGI1 and CASPR2 antibody encephalitis, BCR maturity predominantly is acquired outside the CNS (68). In these patients, CSF is characterized by a notably high frequency of clonally expanded ASC.

CSF memory B cells in NMDAR-E patients exhibited upregulated expression of genes associated with immune regulatory function (*TNFRSF13B* and *ITGB1*), whereas peripheral B cells upregulated genes related to antigen presentation (69). In NMDAR-E patients, CSF B cell subsets followed two differentiation pathways, one leading to the development of memory B cells and the other to plasma cells. Cell–cell interaction networks revealed robust interactions between NMDAR-E CSF and peripheral blood memory B cells and various T cell subsets, including CD4+ T, CD8+ T, and γδT cells, in contrast to control samples. Their findings suggest an immune-regulatory function of memory B cells in the CSF of NMDAR-E patients.

#### 3.1.1 Exploring anti–B cell maturation antigen CAR T cells in NMOSD patients

Anti-CD19/CD20 monoclonal antibody B cell depletion has shown therapeutic efficacy in autoantibody-associated autoimmune diseases, including NMOSD, but a subset of patients remains refractory to current therapies. Thus, chimeric antigen receptor (CAR) T cell therapy is a promising alternative. A single-cell transcriptome, TCR, and BCR data were generated from PBMCs and CSF cells from NMOSD patients treated with BCMA CAR T cells to elucidate the intricacies of this immunotherapy (18). Expanding cytotoxic-like CD8+ CAR T cell clones were pinpointed as the primary agents in autoimmunity. Anti-BCMA CAR T cells with improved chemotaxis successfully traversed the blood-CSF barrier, eradicated plasmablasts and plasma cells in the CSF, and reduced neuroinflammation. The early memory phenotype expressing CD44 in infusion products was potentially linked to CAR T cell persistence in autoimmunity. Additionally, CAR T cells from NMOSD patients exhibited unique characteristics of diminished cytotoxicity compared to those from hematological malignancies, offering mechanistic insights into CAR T cell function in neurological autoimmune diseases. In NMOSD patients, CD8+ CAR T cells exhibited an increased transition to NK-like T cells without significant up-regulation of the exhaustion phenotype. However, high expression of predysfunctional genes in CD8+ CAR T cells in late remission suggests early-stage dysfunctional cells (18).

### 3.2 Neurosarcoidosis

Neurosarcoidosis is a form of sarcoidosis that affects the CNS, causing inflammation and granuloma formation in the brain, spinal cord, and optic nerves (70). The heterogenous nature of the disease poses diagnostic difficulties and has hindered previous efforts to pinpoint common pathophysiological features among patients and to develop targeted treatments. The exact factors that cause neurosarcoidosis to develop in specific tissues of the CNS are not well understood. ScRNA-seq of CSF and blood cells from neurosarcoidosis participants, coupled with T and B cell receptor sequencing, revealed that unlike pulmonary sarcoidosis, which is driven by CD4 T cells, neurosarcoidosis showed an enrichment of CD8 T cell clonal expansion in the CSF (71). These CSF-enriched CD8 T cells were composed of two subsets with differential expression of *EBI2*, *CXCR3*, and *CXCR4*. They also provided evidence that IFN-γ is likely a major bioactive cytokine in neurosarcoidosis compared to other neurologic disorders.

### 3.3 Integrated scRNA-seq analysis of distinct inflammatory diseases

Cantoni and collaborators study analyzed a group of inflammatory diseases of the CNS including myelin oligodendrocyte glycoprotein antibody disease, uveitis, autoimmune encephalitis, and NMOSD (22). They found that these conditions are characterized by a strong increase in Tfh cells in autoimmune encephalitis and NMOSD, supporting B cell activation and autoantibody production (**Table 6**). Unlike MS, CD8+ T cells are not significantly expanded, suggesting that these disorders are more B cell-driven. DC and monocytes are increased in autoimmune encephalitis, but microglia-like cells are not altered, differentiating these diseases from MS. B cells are highly expanded in the CSF, particularly in NMOSD and autoimmune encephalitis, with plasmablasts being significantly more abundant than in MS, reflecting their central role in these diseases.

## 4 Neurodegenerative diseases

All articles related to neurodegenerative diseases are compiled in **Table 3**.

### 4.1 Alzheimer’s disease

AD is a progressive neurodegenerative disorder characterized by memory loss and cognitive decline, with emerging research suggesting that the immune system may play a significant role in its development and progression (72). Amnestic mild cognitive impairment (aMCI) can be considered an early stage of Alzheimer’s disease. It involves cognitive impairment specifically in the domain of learning and memory, including difficulty in retrieving recently stored information. Individuals with aMCI are at higher risk of progressing to AD compared to those with other forms of mild cognitive impairment (73).

#### 4.1.1 Brain-Infiltrating immune cells in Alzheimer’s Disease

##### 4.1.1.1 T cells, mainly CD8+ T cells, infiltrate the brain parenchyma in Alzheimer’s Disease

T cell infiltration in the AD cortex and hippocampus was first described nearly 40 years ago. At that time, it was already evident that CD8+ T cells were significantly more prevalent than CD4+ T cells in both the capillaries and brain parenchyma (74, 75). A subsequent quantitative study determined that 90% of the CD3+ T cells infiltrating the brain parenchyma in AD are CD8+ T cells (76). A higher percentage of GZMA+CD8+ cells were detected in the hippocampi of AD patients compared to control individuals. In AD brains, CD8+ T cells are found in the perivascular space of Aβ+ blood vessels with cerebral amyloid angiopathy, associated with MAP2+ neuronal processes, adjacent to hippocampal Aβ plaques, and in the leptomeninges (77). However, this infiltration significantly correlates with tau pathology but not with amyloid plaques (76). A study quantifying parenchymal and vascular CD8+ T cells found higher numbers in both compartments in AD hippocampi compared to controls. There was a significant positive correlation between parenchymal CD8+ T-cell numbers and Braak stages, but not with vascular CD8+ T cells. A similar trend was observed with Thal stages (78).

Whether the infiltration of CD8+ T cells is a consequence of proteinopathy or precedes it remains unclear. Studies using a mouse model that accumulates expanded and aged CD8+ T cells, along with postmortem brain tissue, suggest that CD8+ T cells may induce both tau aggregation and Aβ plaque formation, leading to neuronal death. Specifically, aging may lead to the accumulation of APP-specific CD8+ T cells with perforin 1 and IFN-γ, exhibiting a TRM phenotype, which could initiate the cascade of neuronal death. In this context, a higher percentage of APP (471–479)/HLA-A2-reactive CD8+ T cells were found in AD tissue compared to controls (79, 80).

The observation of CD3+ T cells expressing the CXCR6 receptor in close proximity to plaque-associated CXCL16+ Iba1+ myeloid cells suggests the involvement of the CXCL16-CXCR6 axis in the infiltration and maintenance of T cells, at least during the clinical stages of the disease (17). An investigation that performed a basic phenotypic characterization of the T cells infiltrating the hippocampal parenchyma (Braak Stages from IV to VI) reported that most T cells found in the parenchyma were CD45RO+ and CD25-, with some being positive for proliferating cell nuclear antigen (PCNA). Additionally, the majority, if not all, of the T cells in the brain parenchyma were negative for the early activation marker CD11b, and there was a slight predominance of CD27- over CD27+ cells (81). A letter study added to the characterization the lack of granzyme B, at least in the Braak stages V and VI, that were the stages included in the study.

##### 4.1.1.2 Neutrophil extracellular traps in Alzheimer’s Disease

In AD patients, the number of neutrophils is significantly higher in both blood vessels and the cortex and hippocampus parenchyma compared to controls. In AD brains, neutrophils migrate into the brain parenchyma, often near amyloid-beta (Aβ) deposits. This is linked to a reduction in endothelial glycocalyx staining in the AD vasculature, potentially leading to increased neutrophil-vascular interactions (82). Neutrophils release neutrophil extracellular traps (NETs) in AD, as evidenced by the presence of citrullinated histones (82, 83). However, the presence of NETs in both compartments (83) versus exclusively in the vasculature (82) remains a subject of debate. NETs are structures formed by neutrophils, composed of DNA, histones, and proteins, that capture and neutralize pathogens. However, excessive NET production can contribute to inflammatory diseases and tissue damage. In this context, experiments conducted with transgenic models of AD suggest that neutrophils may play a deleterious role in the disease. Specifically, depleting neutrophils or preventing LFA1-dependent adhesion has been shown to reduce AD-like neuropathology and improve memory. In these models, neutrophils release NETs and IL-17, further implicating their involvement in disease progression. That said, the exact role of neutrophils in the disease is not entirely clear, but there appears to be a correlation between neutrophil density and proteinopathy. In this context, a transcriptomic analysis and inferred neutrophil presence in the tissue revealed a significantly positive correlation between neutrophil abundance and both the Braak and Thal stages (84). However, these results should be confirmed with histological assessment.

##### 4.1.1.3 CD163+ macrophages in Alzheimer’s Disease

Although several studies suggest cerebral infiltration of monocytes/macrophages, the fact that microglia and macrophages share many markers has made unequivocal demonstration difficult. However, P2Y12 and TMEM119 expression is highly enriched in human microglia and is absent from myeloid cells in blood and CSF. Human microglia are characterized both at the protein and transcriptomic levels by high expression of CD64, CX3CR1, TGF-β1, TREM2, CD115, CCR5, CD32, CD172a, and CD91, and low to absent expression of CD44, CCR2, CD45, CD206, CD163, and CD274 (PD-L1). This contrasts with perivascular macrophages, which are characterized by being CD11b+CD206high CD163+ (85). CD163 is a phagocytic marker of perivascular and meningeal macrophages, and of a subset of peripheral blood monocytes/macrophages believed to be precursors of these brain-resident macrophages (86). The existence of CD163+ monocytes/macrophages in the CSF has also been demonstrated. CD163 immunoreactivity is predominantly restricted to perivascular macrophages in the brains of most control cases. However, in Braak 5/6 AD cases, CD163+ microglia-like cells are found in the parenchyma of the frontal and occipital cortices. These parenchymal cells do not express CD206, and a significant proportion lack Iba1 expression. They are associated with amyloid-beta (Aβ) plaques but do not interact with intracellular neurofibrillary tangles. The presence of these cells near areas of blood-brain barrier damage suggests that they may have infiltrated from outside the parenchyma. Additionally, these CD163+ cells exhibit CD68 positivity, particularly in cells with a more amoeboid morphology, indicating enhanced phagocytic activity. It is highly likely that these macrophages contribute to the clearance of debris resulting from neuronal death (87). A CyTOF-based study supports this hypothesis. In this study, infiltrating macrophages in the AD gyrus frontalis medialis exhibited an enhanced phagocytic phenotype, whereas choroid plexus macrophages and parenchymal microglia showed a downregulation of specific markers indicative of a reduced phagocytic capacity (88).

#### 4.1.2 CSF Single-cell transcriptomics in Alzheimer’s Disease

##### 4.1.2.1 CD8+ T cells relevance in Alzheimer’s Disease

The first scRNAseq study that included CSF from patients AD, underscored the significance of CD8+ T cells patrolling the CSF (77). This study demonstrated that approximately half of the highly expanded clones in MCI and AD individuals were CD8+ TEMRA cells, which exhibited increased expression of cytotoxic effector genes, including *NKG7* and *GZMA*, as well as *HLA-C* and beta-2-microglobulin. Additionally, they reported a patient with AD who exhibited a remarkable expansion of a CD8+CD45RA+CD27− TEMRA clone, comprising 44% of all CD8+ TCRs. A subsequent study that reanalyzed the data from this article, focusing on specific samples, confirmed that CD8+ TEMRA cells are the most expanded T cell subtype, followed by effector memory CD8+ T cells in AD (89).

Despite the evident role of neutrophils in AD, their presence does not appear to be manifested in the CSF. In fact, although there is an increase in markers of neutrophil activation in the blood of both AD patients and individuals with MCI, this increase cannot be found in the CSF (90).

##### 4.1.2.2 CD8+ T cells CXCL16-CXCR6 axis in aging and Alzheimer’s Disease

A single cell analysis including 45 cognitive normal individuals aged 54–82 years and 14 individuals with AD or mild cognitive impairment revealed both changes associated to aging and AD (17). Non-classical monocytes showed a strong reduction in expression with age of cytokine genes such as *CCL3*, *CCL4*, *TNF* and *IL1B*. This was accompanied by increased expression of genes involved in lipid transport, including APOE, APOC1, and higher expression of PLTP which is linked to inflammation (91).

An age-related increase in *CD74* expression was observed among CD4+ and CD8+ T cells. *CD74* encodes the HLA class II histocompatibility antigen gamma chain, a marker of T cell activation. Additionally, CD4+ and CD8+ T cells showed upregulation of genes encoding the granzyme family of serine proteases. Specifically, *GZMH* was upregulated in CD8+ T cells, and *GZMH* and *GZMM* were upregulated in CD4+ T cells (17). When compared to cognitively impaired individuals, a downregulation of lipid transport genes in monocytes was observed, along with altered cytokine signaling to CD8 T cells. Additionally, clonal CD8 T effector memory cells showed upregulation of *CXCR6* and its ligand, CXCL16, was found to be elevated in the CSF in cognitively impaired subjects. Cell-cell interactions analysis of CXCL16-CXCR6 signaling in cognitively impaired CSF indicated non-classical monocytes as the primary source of CXCL16 for CXCR6 expressed on CD8+ T cells (17). Altogether suggests that CXCL16-CXCR6 signaling may facilitate antigen-specific T cell entry into the brain in cognitively impaired individuals. It is important to note that the TCR repertoire of cognitively impaired subjects closely resembles that of advanced-aged individuals.

#### 4.1.3 Flow cytometry studies also suggest a pathogenic role of CD8 T cells in Alzheimer’s Disease

A flow cytometry-based study revealed an increase in activated CD8+ and CD4+ T cells in the CSF of mild AD and MCI patients, with CD8+ T cell activation linked to neuropsychological deficits and parahippocampal damage (92). No changes in T cell frequencies were noted in AD patients with impaired daily activities, but a reduction in TCR diversity among CD4+ T cells indicated greater clonal expansion (93). CD4+ T cell frequencies decreased significantly in severe AD (94), consistent with observations in aMCI patients with amyloid-beta (Aβ) accumulation (95, 96). Both AD and MCI patients showed a shift from central memory to late-stage effector T cells, with an increase in naive CD8+ T cells (94). Increased CD8+ T cells in aMCI patients with higher Aβ burden suggest a pathogenic role (95). Opposing roles of CD4+ and CD8+ T cells in Aβ deposition were noted. Increased expression of *CD139, TREM2*, and *CD33* in CSF DN T cells suggests an anti-inflammatory effect of these cells in AD (88). Conversely, NKT and NK cells in the CSF were correlated with cognitive impairment and phosphorylated tau levels (94).

### 4.2 Neuronal synucleinopathies: Parkinson’s Disease and dementia with Lewy bodies

Neuronal synucleinopathies, such as PD and dementia with DLB, are neurodegenerative disorders characterized by the abnormal accumulation of α-synuclein protein in neurons (97). PD primarily affects motor function, while DLB is associated with both cognitive decline and motor symptoms. Recent research underscores the significant role of the immune system in these diseases.

#### 4.2.1 Brain-infiltrating immune cells in PD

##### 4.2.1.1 CD8+ T cells infiltrate brain parenchyma in clinical Parkinson’s Disease and early premotor stages

The infiltration of CD8 T cells into the substantia nigra in PD was first described, as in AD, four decades ago, although this observation was based on a single case and lacked quantitative analysis (98). Recent quantitative studies have confirmed that in PD, there is a prominent infiltration of CD8 T cells into the brain parenchyma, specifically in the substantia nigra (99–101). One of these studies demonstrated that CD8+ T cells infiltrate the brain parenchyma of the substantia nigra and establish contact with dopaminergic neurons during the very early premotor stages of PD (99). This infiltration precedes α-synuclein aggregation and neuronal loss, implicating these cells in the early pathogenesis of the neuronal death cascade. CD8+ T cells in this context exhibit a tissue-resident phenotype and possess a full cytotoxic arsenal, including various granzymes such as granzyme B, and IFN-γ. In the clinical motor stages of PD, a positive correlation is observed between the density of CD8+ T cells and neuronal loss, with persistent interactions noted between CD8+ T cells and dopaminergic neurons in the substantia nigra (99). These findings suggest that PD may have an infectious or autoimmune origin, or possibly a combination of both (102). However, the nature of the antigen that induces this response remains unknown. We hope that antigen prediction based on TCR sequences will contribute to this endeavor.

Although CD4+ T cell infiltration is not evident, an increase in perivascular CD4+ T cells is observed in some cases during later stages (clinical PD), suggesting a role for these cells in compensatory mechanisms, potentially involving humoral responses against protein aggregates (99). Notably, while B cells are absent in the affected brain regions in PD, the deposition of antibodies in neurons containing Lewy bodies has been documented (103).

##### 4.2.1.2 CD163+ macrophages in Parkinson’s Disease postmortem tissue

In the specific case of neuronal synucleinopathies, including PD, Parkinson’s disease dementia (PDD), and dementia with Lewy bodies (DLB), there is an observed increase in the density of perivascular macrophages expressing CD163. Additionally, CD163+ cells have been detected in the brain parenchyma. Similar to AD, their close proximity to damaged blood-brain barrier, suggests that these cells have infiltrated from outside the brain. These cells exhibit the same phagocytic phenotype as those found in AD. In this context, microglia were occasionally found adjacent to extracellular α-synuclein (87).

#### 4.2.2 CSF single-cell transcriptomics in Parkinson’s Disease

##### 4.2.2.1 CXCR4-CXCL12 axis is involved in T cell infiltration

To date, only one study has generated scRNAseq data to evaluate changes occurring in synucleinopathies, which includes scTCRseq data. This study encompasses cases of PDD and DLB, both considered types of Lewy body dementia (LBD). PDD is regarded as a later stage of PD when the cerebral cortex is affected (104). The study identifies nine cell clusters, including one annotated as CD4+ T cell, two as CD8+ T cells, specifically CD8+ T EM and CD8+ Granulysin+, and one as a double-negative (DN) T cells (19). The most differentially expressed genes are observed in CD4+ and CD8+ T cells when comparing the pathological cases group with the controls. Both subtypes of CD8+ T cells exhibit significantly more clonal expansion compared to CD4+ T cells in both controls and the LBD group. However, no changes are observed in the degree of expansion in any of the T cell subtypes when comparing both groups at the T cell resolution that they reached. In the study, the focus is exclusively on CD4+ T cells, particularly those with clonal expansion. No additional analyses were conducted on CD8+ T cell populations. Pathway analysis of differentially expressed genes in the expanded CD4+ T cells revealed that regulation of cytokine-mediated signaling and intracellular signal transduction were the most altered pathways, involving CXCR4. The study also uncovered CD4+ T cell populations unique to the CSF, identifying upregulated CXCR4, CD69, and TSC22D3 as the primary genes defining these unique CSF T cells, suggesting that CXCR4 may regulate the homing of CD4+ T cells to the LBD brain. Additionally, higher levels of CSF CXCL12 were observed in PD, with CXCL12 levels correlating most positively with neurofilament light chain in PDD. They proposed that dysregulated CXCR4-CXCL12 signaling is associated with neurodegeneration in LBD. They also reported that the expression of Killer cell lectin-like receptor subfamily B, member 1 (KLRB1), a marker of Th17 memory CD4+ T cells and Tc17 CD8+ T cells, was upregulated. Studies that include cases of PD without dementia will determine whether the changes described in this study are exclusive to PDD or are common to all stages of PD. Although T cells were shown to infiltrate the brain parenchyma and were found in proximity to neurons with α-synuclein aggregates (19), it was not clarified whether these were CD4+ or CD8+ T cells.

##### 4.2.2.2 Reanalysis of the original dataset: RAC1 and cell trajectories

The reanalysis of the data of the original paper annotated 10 subtypes of cells, including CD4+, naive CD4+, CD8+, and cycling T cells, among the T cell populations (105). They also annotated a cluster of NK cells, as in the original study, along with other cell types. They found that CD8+ T cells were the most transcriptionally dysregulated immune cell subtype. They determined that the Rac family small GTPase 1 (RAC1), involved in cell migration and immune response, was differentially expressed in CD8+ T cells, CD4+ T cells, DC, NK cells, and macrophages. RAC1 was the most significantly upregulated gene in NK cells from PDD patients, and the ratio of NK cells was found to be increased in the CSF of PD patients. Although one article claims that NK cells are present in the human substantia nigra much more prominently in cases of PD and DLB than in control cases, the marker they used, CD244, is not exclusive to NK cells and is also expressed on other immune cells, such as CD8+ T cells (105).

Another reanalysis of the original dataset integrated with their own blood dataset implemented trajectory analysis (106). The combined expression and TCR-based lineage tracking revealed a significant population of CD8+ T cells progressing from central memory to terminal effector states in PD patients. Additionally, a notably expanded group of cytotoxic CD4+ T cells was identified, derived from Th1 cells through TCR-based fate decisions.

#### 4.2.3 Flow cytometric analysis of T cell activation and other findings

Among the various neuronal synucleinopathies, flow cytometry studies have only been conducted with CSF from PD patients. These studies are scarce and have utilized very few markers. Nevertheless, activation of both CD4+ T cells and CD8+ T cells has been demonstrated, with the latter showing a higher proportion of activated cells (HLA-DR+) (107). An increase in γδT cells has also been reported, although it was not determined whether these were DN or CD8+ T cells (108). γδT cells are one of the cellular subtypes that do not manifest according to the clustering and annotation methods employed to identify different cellular subtypes. In this context, the original scRNA-seq study which included cases of LBD, did not annotate this cellular subtype (77). A CSF flow cytometry study, reported a shift from classical monocytes (CD14+/CD16−) to non-classical monocytes (CD14+/CD16+) (107). Additionally, another study found an increase in the frequencies of both populations (109). No differences were observed in the proportions or absolute numbers of granulocytes and B cells (107).

#### 4.2.4 Integrated scRNA-seq analysis of Alzheimer’s Disease and neuronal synucleinopathies

Cantoni and collaborators reanalyzed data from AD, PD, and dementia with DLB, along with MCI as a prodromal stage of AD, grouping them collectively as neurodegenerative diseases (**Table 6**) (22). These conditions share common neuroinflammatory features but exhibit distinct immune alterations. Neurodegenerative diseases are characterized by an increase in Th1 cells in the CSF, suggesting a persistent inflammatory state. Additionally, there is an enrichment of CD8+ effector memory T cells expressing granzyme B in the CSF. These cytotoxic T cells may contribute to neuronal death as some studies with postmortem brain tissue suggest (99, 102). A notable feature of neurodegenerative diseases is the increase in FN1+ and SPP1+ microglia-like cells, which express risk genes associated with AD and PD. Unlike MS and other inflammatory diseases, CD16+ monocytes are not elevated, suggesting that inflammation in neurodegenerative diseases is primarily driven by resident immune cells. While B cells are not significantly expanded, some patients with AD and DLB exhibit a mild increase in plasmablasts, hinting at a subtle humoral immune component (22).

### 4.3 Amyotrophic lateral sclerosis

Amyotrophic lateral sclerosis (ALS) is a disease marked by progressive motor neuron loss, muscle weakness, and respiratory failure. While the exact mechanisms remain unclear, there is growing consensus on the immune system’s involvement in ALS. Neuroinflammation, including glial activation, T cell infiltration, and systemic immune activation, is commonly observed in ALS (16). A single-cell analysis showed increased amounts of cytotoxic CD4+ T cells and CD4+ T cells with an activated phenotype (CCR7− CCL5+) but decreased amounts of monocytes and CD4+ CD8+ double-positive T cells, in the CSF of ALS patients compared with controls (110). Focusing on genes differentially expressed in the T cell subsets, they found an overexpression of set of genes that control for cellular cytotoxicity, including *GNLY, GZMA, GZMB, GZMH, GZMK*, *PRF1, CTSW, KLRB1, KLRD1*, and *NKG7*. ALS patients exhibited greater levels of TCR expansions in cytotoxic CD4+ T cells as well as in CD4+ and CD8+ T cells with an activated phenotype (CCR7− CCL5+), compared with controls. Those subsets and γδT cells contained hyperexpanded clones in ALS. Their results suggest that the strength and nature of TCR recognition during ALS promote the differentiation of antigen-specific Eomesodermin-expressing cytotoxic CD4+ T cells and Th1 cells. Overall, the findings highlight the significance of T cell immunity in ALS pathogenesis.

### 4.4 Spinal muscular atrophy

5q-associated spinal muscular atrophy (SMA) is a genetic neuromuscular disorder caused by mutations in the SMN1 gene located on chromosome 5q (111). Adaptive immunity may play a role in SMA, similar to other motor neuron diseases such as ALS, although the mechanisms remain unclear. A study involving SMA patients identified an expansion of cytotoxic NK cells and CD8+ T cells in the CSF and near chromatolytic motoneurons in untreated patients, suggesting their role in the pathogenesis of the disease (112). Transcriptomics and histology suggest these lymphocytes are recruited and activated by IL-18-secreting myeloid cells in the CNS.

## 5 Narcolepsy

Narcolepsy is a chronic neurological disorder characterized by excessive daytime sleepiness and sudden sleep attacks, with two main types: Type 1 (NT1), which includes cataplexy, and Type 2 (NT2), which does not (113). NT-1 is characterized by the decreased levels of orexin-A due to the loss of the neurons that produce it. The exact cause of narcolepsy remains unclear, but the significant HLA association (HLA-DQB1*06:02 allele) and elevated levels of CD4+ T cells that react to orexin-A in peripheral blood indicate potential autoimmune activity in the hypothalamus (114). Only one article has been published that has conducted CSF single-cell transcriptomics (**Table 3**). This study compared the cellular landscapes of CSF cells from patients with narcolepsy type 1 (NT1), narcolepsy type 2 (NT2), MS, and idiopathic IIH to identify differences in gene expression levels (115). The study revealed that the CSF cell composition and gene expression levels in narcoleptic patients are more similar to those in IIH, a non-inflammatory disease, than to those in MS. The most notable finding is that *MTRNR2L12* and *MTRNR2L8* which encode peptides homologous to the mitochondria-encoded peptide Humanin, were not significantly detected in CD4+ and CD8+ T cells from MS and IIH patients, yet they were the most striking differentially expressed genes in NT1 and NT2. Humanin is known for its anti-apoptotic effects (116), but the role of its homologs in the pathogenesis of narcolepsy remains to be fully understood. One possible effect of these Humanin homologs is the dysregulation of the immune response due to their anti-apoptotic effect on T cells.

## 6 Neurological infectious diseases

All articles related to neurological infectious diseases are compiled in **Table 4**.

Cantoni and collaborators study compared several neurological infectious diseases with controls. Infectious neurological diseases included viral encephalitis, Neuro-COVID, and HIV-associated neurologic disease (22). These conditions are marked by strong innate immune activation and altered T cell responses. Viral infections elicited a strong Th1 response, particularly in viral encephalitis and HIV-associated neurological disease (**Table 6**). There was also an increase in interferon-producing Th cells. CD8+ HLA-DR+ T cells, which are activated cytotoxic T cells, were significantly reduced in viral encephalitis and HIV-associated neurological disease, suggesting immune exhaustion. These diseases were characterized by a strong increase in CD16+ monocytes, LAMP3+ DC, and neutrophils in the CSF, indicating a robust inflammatory response. A striking expansion of plasmablasts in the CSF was observed, particularly in viral encephalitis and HIV-associated neurological disease, reflecting an active antibody response.

### 6.1 Neuro-COVID

Neuro-COVID refers to a wide variety of neurological manifestations observed in COVID-19 patients, which can include headaches, seizures, encephalopathy, and stroke (117). Understanding the immune response in Neuro-COVID is crucial for developing effective diagnostic and therapeutic strategies.

A study comparing CSF immune cell profiles in Neuro-COVID patients to those with non-inflammatory neurological conditions, autoimmune diseases, including MS, and viral encephalitis, revealed an expansion of dedifferentiated monocytes and exhausted CD4+ T cells in Neuro-COVID patients (118). Although an interferon response was present, it was significantly weaker compared to viral encephalitis, suggesting an impaired antiviral immune response. TCR repertoires indicated broad clonal expansion of T cells in severe Neuro-COVID cases, alongside a curtailed IFN response, contributing to immune dysfunction. Severe Neuro-COVID showed widespread immune dysregulation, with increased expression of exhaustion markers such as *PD-1, CTLA-4*, and *TIM-3* in T cells, while milder cases had a more balanced immune response. The study suggests immune profiling of CSF could serve as a diagnostic tool for Neuro-COVID and explores the potential of immune checkpoint inhibitors as a therapeutic option (118).

Another study which included CSF and blood samples from Neuro-COVID patients and healthy controls revealed compartmentalized T cell activation in the CNS, with distinct immune responses in CSF compared to the periphery (119). CSF T cells showed increased activation and expansion, suggesting a localized immune response to SARS-CoV-2. All COVID-19 patients had anti-SARS-CoV-2 antibodies in their CSF, with target epitopes differing from those in the blood, indicating a unique humoral response within the CNS. Additionally, five out of seven patients had antineural autoantibodies in their CSF, suggesting a potential role for autoimmunity in COVID-19-related neurological symptoms.

### 6.2 Neurological HIV

HIV infection has long been associated with various neurological complications, even in the era of effective antiretroviral therapy. The CNS often becomes a target for HIV, leading to immune activation and potential neuronal damage. This can result in a range of neurocognitive impairments, from mild cognitive dysfunction to more severe conditions such as HIV-associated neurocognitive disorders (120). Understanding the mechanisms behind these neurological effects is crucial for developing targeted therapies. By using scRNA-seq on CSF and blood from adults with and without HIV, researchers identified a rare subset of myeloid cells (less than 5% of cells) that are unique to CSF. These cells show a gene expression pattern that significantly overlaps with microglia associated with neurodegenerative diseases (121). This subset is characterized by high expression of 60 distinct genes when compared with all other myeloid subsets. Several of these genes are produced in the CNS almost exclusively by microglia, including *C1QA–C* and *TREM2* (122, 123). Altogether, these findings suggest that microglia-like cells may be contributing to neural injury during HIV infection. Consistent results were found in a patient with MS, infected with HIV (124).

### 6.3 Progressive multifocal leukoencephalopathy

Progressive multifocal leukoencephalopathy (PML) is a rare and serious brain infection caused by the JC virus (125). The JC virus, which is usually harmless in people with healthy immune systems, can cause PML in individuals with weakened immune systems, such as those with HIV/AIDS, leukemia, lymphoma, or those undergoing immunosuppressive therapy. Single-cell transcriptomics of CSF cells reveal an enrichment of distinct CD4+ and CD8+ T cells expressing chemokine receptors *CCR2*, *CCR5*, and *CXCR3*, along with *ITGA4* and genetic PML risk genes *STXBP2* and *LY9* (126). Besides general inflammatory chemokines like CCL5 and CXCL10, the CSF of PML patients specifically contains CCL2 and CCL4. All together indicates that specific immune cell subpopulations migrate into the central nervous system to combat PML, and their absence may be associated with PML development. Monitoring these cells could provide insights into PML risk, and enhancing their recruitment or function before therapeutic immune reconstitution might improve the risk-benefit ratio.

### 6.4 Bacterial meningitis

scRNA-seq, supplemented by bulk transcriptome sequencing, was employed to elucidate the characteristics of CSF cells during bacterial meningitis progression in children involving distinct bacteria (127). This analysis identified 18 distinct immune cell clusters within the CSF, comprising two neutrophil subtypes. Notably, there was a decrease in myeloid cell proportions and an increase in lymphoid cell proportions as the disease progressed. Two novel subtypes were identified: FFAR2+TNFAIP6+ neutrophils and THBS1+IL1B+ monocytes. The quantities of these subtypes positively correlated with the intensity of the inflammatory response in the CSF. CSF from bacterial meningitis patients with therapeutic failure showed distinct cell heterogeneity, including altered intercellular communications and increased proportions of type II myeloid DC and plasmacytoid DC. These findings indicate that specific immune cell subpopulations and their dynamics are crucial in bacterial pathogenesis and treatment response, suggesting potential targets for therapeutic intervention and biomarkers for disease monitoring.

### 6.5 Tuberculous meningitis

Tuberculous meningitis is a severe manifestation of tuberculosis that leads to significant mortality and neurological impairment (128). The pathogenesis of this meningitis involves complex immune dysregulation, yet the precise immune mechanisms remain poorly understood. scRNA-seq was performed on PBMCs and CSF cells isolated from six children with tuberculous meningitis and discovered that complement-activated microglia-like cells are associated with a neuroinflammatory response that leads to persistent meningitis (129). Consistently, increased levels of complement protein (C1Q), inflammatory markers (C-reactive protein), and inflammatory factors (TNF-α and IL-6) were observed in CSF cells but not in blood. Microglia-like cells were found to recruit CD4+ TRM, with Th1 and Th17 phenotype, through CXCL16/CXCR6. All together suggests this microglia-like subset activates complement and interact with the CD4+ TRM cell subset to amplify inflammatory signals, potentially contributing to hyperinflammation and an immune response in infected tissues.

### 6.6 Subarachnoid neurocysticercosis

Subarachnoid neurocysticercosis is a form of neurocysticercosis, which is an infection of the CNS caused by the parasite *Taenia solium* (130). The relative increase of IL-10 (immunosuppressive) compared to IL-12 (pro-inflammatory) is associated with a longer duration of treatment (131). A single-cell study has demonstrated that the major source of IL-10 is induced regulatory T cells, and to a lesser extent, natural regulatory T cells and Th17 cells (132).

## 7 Brain cancer and metastases

All articles related to brain cancer and metastases, including leptomeningeal metastases, are compiled in **Table 5**.

The complexity of brain structures and the diverse characteristics of tumors make it difficult to obtain tissue samples using conventional biopsy techniques, which are invasive and carry significant risks. Single-cell transcriptomics offers a noninvasive approach to analyzing the tumor immune microenvironment not only of brain tumors but also brain metastases. In addition, tumor circulating cells can be analyzed. This approach enables the identification of distinct cell populations, their gene expression profiles, and the molecular pathways involved in tumor progression and immune evasion. By leveraging single-cell analysis, researchers can uncover novel therapeutic targets and develop more effective, personalized treatment strategies for brain tumors and metastases.

### 7.1 Glioblastoma

Glioblastoma is an aggressive brain tumor marked by rapid growth and high invasiveness, often resulting in poor prognosis despite existing therapeutic interventions. Therefore, investigating the mechanisms of tumor invasion is crucial. In this context, a significant enrichment of Tregs was observed in the CSF of a patient with malignant glioblastoma compared to a patient with non-malignant brain cancer (133). This suggests that Tregs contribute to the inhibitory microenvironment necessary for tumor recurrence. Additionally, macrophages and neutrophils were notably abundant in malignant CSF, reinforcing their role in shaping the tumor microenvironment. A key discovery of the study is the identification of S100A9 as a potential biomarker for glioblastoma recurrence, with its expression significantly upregulated in malignant CSF. Moreover, CD8+ T cells, while present, exhibited signs of exhaustion, reducing their effectiveness in combating tumor cells.

### 7.2 Leptomeningeal metastases

Leptomeningeal metastatic disease occurs as a result of cancer cells spreading from extracranial and specific intracranial malignancies into the leptomeninges and cerebrospinal fluid (134). The use of single-cell transcriptomics to investigate the CSF in patients with brain metastases and leptomeningeal metastases (LM) has provided significant insights into the tumor microenvironment and immune landscape. Immune cell profiling of breast metastasis using scRNA-seq and scTCR-seq found variable immune infiltration patterns. High immune infiltration and strong IFN-γ signatures correlated with prolonged survival in lung adenocarcinoma patients. CSF immune profiles mirrored breast metastasis immune landscapes, suggesting CSF profiling as a proxy for brain tumor immune status (20). The analysis of CSF from 12 breast cancer patients with breast metastases found that patients with better prognosis had more T cells and DC, which support anti-tumor immunity, while immunosuppressive cells like tumor-associated macrophages were less abundant. Gene expression analysis identified markers regulating immune activity, with genes such as *CCR5, LYZ*, and *IGKC* promoting tumor immune function, while *SCGB2A2* and *CD24* inhibited it (135). A previously published scRNA-seq analysis of CSF from LM patients with breast cancer or lung cancer revealed significant immune alterations, particularly in macrophages and Tregs, indicating also an immunosuppressive microenvironment. The study identified five molecular subtypes of CSF circulating tumor cells from breast cancer LM patients and highlighted the strong immunosuppressive crosstalk between macrophages and circulating tumor cells (136). Analysis of circulating tumor cells from the CSF of lung adenocarcinoma LM patients using scRNA-seq showed upregulated metabolism-related and cell adhesion pathways essential for tumor survival, significant heterogeneity among circulating tumor cells, partial epithelial-to-mesenchymal transition, cancer stem cell properties, and immune evasion markers. These findings provide insights into metabolic adaptation, immune evasion, tumor heterogeneity, and metastatic potential of CSF-circulating tumor cells (137). The tumor microenvironment in central nervous system metastases, particularly LM, was shown to involve cancer cells in the CSF expressing the iron-binding protein lipocalin-2 and its receptor *SCL22A17*. This transcriptional diversity gives cancer cells a competitive edge over macrophages, which do not produce lipocalin-2, thereby depleting macrophages of iron and impairing their respiratory burst and phagocytic functions (138). Exploration of the role of CEACAM6 in non-small cell lung cancer LM using RNA analysis of CSF identified CEACAM6 as highly expressed in tumor-associated cell-free RNA. Functional experiments demonstrated that CEACAM6 enhances non-small cell lung cancer cell migration, suggesting its potential as a non-invasive biomarker for LM diagnosis (139).

#### 7.2.1 Treatment response in leptomeningeal metastases

Single-cell transcriptomics can also be useful for studying treatment responses. Investigation of genomic and transcriptomic features associated with immunotherapy response in LM patients found that immune checkpoint inhibitors initially increased CD8+ T cell activity, but this response was transient due to immune evasion mechanisms. The study highlighted the need for combinatorial therapeutic strategies (140). The investigation of the immune microenvironment of melanoma brain metastases and LM using scRNA-seq found distinct immune landscapes, with melanoma brain metastases samples showing diverse immune infiltration with active T cells, while LM samples exhibited severe T cell dysfunction and immune suppression. An exceptional LM survivor responded well to PD-1 therapy, displaying higher T cell and DC levels (141). More recently, a study highlighted an immunosuppressive lipid-associated macrophage subtype involved in osimertinib resistance and LMs, suggesting a potential therapeutic target (139).

### 7.3 CNS lymphomas

Single-cell transcriptomics has also been used to investigate the immune landscape and cellular heterogeneity in CNS lymphomas (142). A study revealed significant heterogeneity among CSF-diffuse large B cells, including variations in cell cycle states, cancer-testis antigen expression, and transcriptional subtypes. Key findings include the upregulation of genes like *EZH2, PHF19*, and *RRM2*, and the presence of both kappa (κ) and lambda (λ) light chains in some cells. The study suggests that CSF- diffuse large B cells can serve as a non-invasive diagnostic tool, highlighting tumor-specific gene expression patterns and immune evasion mechanisms (143). Another study investigated intratumor heterogeneity and T cell exhaustion in primary CNS lymphoma using scRNA-seq, B cell receptor sequencing, and spatial transcriptomics on biopsy-derived cells, peripheral blood, and CSF (144). Findings revealed significant transcriptional and spatial heterogeneity in malignant B cells, with hyperexpanded clones in the biopsy and CSF, but not in the blood, suggesting CNS origin. The tumor microenvironment showed widespread T cell exhaustion with upregulated immune checkpoint molecules (*PD-1, CTLA-4, TIM-3, TIGIT, LAG-3*) and checkpoint ligands on malignant B cells. The relapsed clone exhibited increased CCR7 expression, indicating potential CNS spread mechanisms. These findings suggest checkpoint inhibitors targeting *TIGIT, TIM-3*, and *PD-1* as promising therapies for primary CNS lymphoma, highlighting the need for personalized treatments based on the heterogeneity of malignant cells.

Primary central nervous system lymphomas anaplastic large cell lymphoma are very rare non-Hodgkin’s lymphomas. In a case with this condition, scRNAs-seq identified 45 genes upregulated in CSF anaplastic large cells compared to normal T cells (145). These genes highlighted pathways for cell proliferation and metabolism, and a loss of immune identity. The heterogeneity of these cells was demonstrated by the expression of cancer-testis antigens and cell-cycle genes. Transcription factors EZH2 and NFYC were upregulated in CSF-anaplastic large cells. The patient achieved complete remission after four cycles of chemotherapy with methotrexate and intrathecal cytarabine. This study provides new insights into the diagnosis and mechanisms of this lymphoma.

## 8 Conclusion and future directions

The first single-cell transcriptomics article was published in 2009 (146), but it was not until nine years later that the first article involving CSF was published (60). In these few years, several articles have emerged, offering new insights into neurological diseases. For instance, they have highlighted the significant role of CD8+ T cells with specific signatures in diseases such as MS, AD, and PD. Specifically, in MS, these studies have established the importance of a dysfunctional antiviral response in the disease’s pathogenesis. Additionally, they have helped identify new therapeutic targets and better understand the mechanisms of immunotherapies in different neurological diseases. However, despite its transformative impact, several limitations persist in the field, hindering its full potential. These limitations arise from various factors. The technology itself is complex and involves multiple steps at both the wet lab and bioinformatics analysis levels, each of which can introduce artifacts (147). Therefore, it is crucial to establish comprehensive quality criteria to ensure that deviations from reality are minimized at each step. This includes ensuring appropriate sequencing depth and cell coverage and developing bioinformatics pipelines that make minimal assumptions and yield biologically relevant results.

scRNA-seq in general has several limitations and challenges (148), while scRNA-seq in CSF has its own unique constraints. CSF typically contains a sparse population of cells, making it difficult to obtain sufficient numbers for comprehensive analysis. This limitation can be exacerbated if the cell recovery rate of the employed methodology is low due to inefficient cell capture and cell loss during processing (149). This limitation can lead to biased results and reduced statistical power. The risks associated with lumbar puncture have resulted in the exclusion of healthy controls in many studies. Instead, cases of IIH are included, when possible. This, along with high costs, has resulted in modest patient numbers in many studies, limiting patient stratification. In many cases, prodromal cases of a disease have been combined with the disease itself, preventing the differentiation of initiating mechanisms from disease progression mechanisms and compensatory mechanisms that occur in later stages. To mitigate this difficulty, the practice of integrating different datasets has been extended, using tools aimed at minimizing batch effects. However, these tools are not exempt from generating artifacts, as they can minimize real biological differences (150).

Technical variability is a significant concern in scRNA-seq. Variations in sample handling, cell capture efficiency, and sequencing depth can introduce noise and affect the reproducibility of results (10, 151). Ensuring consistent protocols and rigorous quality control measures is essential to mitigate these effects. Bias in transcript coverage often favors the detection of highly abundant transcripts while underrepresenting low-abundance ones, skewing the interpretation of gene expression profiles. The efficiency of capturing single cells and their RNA content can be low, leading to incomplete or missing data (152), which results in the loss of rare cell types and critical information about cellular heterogeneity. Compared to bulk RNA-seq, scRNA-seq data are more prone to technical noise, with factors such as amplification bias, dropout events, and sequencing errors complicating data analysis and interpretation.

Computational challenges are also prevalent in scRNA-seq. Analyzing the data requires sophisticated computational tools and methods, including normalization, batch effect correction, and dimensionality reduction, which are complex and computationally intensive (10, 151). Developing robust algorithms that can handle the high variability and complexity of scRNA-seq data remains an ongoing challenge. Furthermore, the cost of scRNA-seq can be prohibitive, especially for large-scale studies. Scaling up experiments to include thousands or millions of cells requires substantial computational resources and data storage capabilities. Addressing these limitations and challenges is crucial for advancing the field of single-cell analysis and unlocking its full potential in understanding complex biological systems.

In the case of immune cells, particularly T cells, their plasticity presents a challenge, as transitional cells between two subtypes complicate clustering. Fortunately, tools have been developed to analyze from this perspective (153). The fundamental issue in single-cell transcriptomics is the occurrence of dropouts (154), which can affect critical transcripts such as CD4, making it difficult to discern whether a T cell is DN or CD4+.

Regarding the study of TCRs and clonal expansion, the expected conclusive results have not yet been achieved. However, important insights have been gained, such as the observation that clones infiltrating the brain can also be found in the CSF, and that clonal expansion in the CSF is not necessarily indicative of pathology. Additionally, while antigenic prediction holds extraordinary potential, it requires more comprehensive databases and rigorous experimental validation to be fully effective (155).

Single-cell transcriptomics is continually advancing, with enhanced experimental designs and bioinformatics tools being developed through the application of deep learning (156). Reaching a consensus on the optimal methods for analyzing CSF cells and identifying cellular subsets under both physiological and aging conditions is essential. This will improve our understanding of the changes that occur in various neurodegenerative diseases.
